# Effects of Comorbidities on Pain and Function After Total Hip Arthroplasty

**DOI:** 10.3389/fsurg.2022.829303

**Published:** 2022-05-11

**Authors:** Pingwen Lan, Xi Chen, Zhi Fang, Jianjun Zhang, Shuping Liu, Yuehong Liu

**Affiliations:** Department of Orthopedics, People’s Hospital of Deyang City, Deyang, China

**Keywords:** total hip arthroplasty, comorbidity, readmission, complications, elderly

## Abstract

**Background:**

The growing number of patients undergoing total hip arthroplasty (THA) and postoperative outcomes receive increasing attention from doctors and patients. This study aimed to elucidate the effects of comorbidities on postoperative function, pain, complications, readmission rate, and mortality.

**Methods:**

We included consecutive patients who underwent primary unilateral THA between 2017 and 2019. The Charlson comorbidity index (CCI) and the WOMAC and SF-36 (physical function, body pain) scales were assessed preoperatively and at 3, 6, 12, and 24 months postoperatively. The complications, 30-day readmission, and mortality rates assessed the impact of comorbidities and their changes over time on the WOMAC and SF-36 scores during follow-up. We used mixed model linear regression to examine the association of worsening comorbidity post-THA with change in WOMAC and SF-36 scores in the subsequent follow-up periods, controlling for age, length of follow-up, and repeated observations.

**Results:**

This study included 468 patients, divided into four groups based on comorbidity burden (CCI-0, 1, 2, and ≥3). The physiological function recovery and pain scores in the CCI ≥ 3 group were inferior to the other groups and took longer than the other groups (6 vs. 3 months) to reach their best level. The four groups preoperative waiting times were 2.41 ± 0.74, 2.97 ± 0.65, 3.80 ± 0.53, and 5.01 ± 0.71 days, respectively. The complications, 30-day readmission, and 1-year mortality rates for the overall and the CCI ≥ 3 group were 1.92% and 4.69%, 0.85% and 2.01%, and 0.43% and 1.34%, respectively, with no mortality in the other groups.

**Conclusion:**

Patients with higher CCI were more susceptible to physical function and pain outcome deterioration, experienced longer waiting time before surgery, took longer to recover, and had higher rates of complications, 30-day readmission, and mortality after THA. Older age in the group led to a greater impact.

## Introduction

Total hip arthroplasty (THA) is a safe, successful, and economical treatment for advanced hip osteoarthritis. It relieves pain and improves patient function and quality of life (1–5). These procedures have increased in numbers worldwide over the last few decades (6, 7) and are expected to further surge due to the increase in life expectancy and osteoarthritis prevalence (8, 9).

Approximately 1.2 million THAs are performed worldwide each year (10). The rapid increase in numbers could be attributed to the rise in population age, increase in arthritis prevalence, and other factors that create the need for the procedure (11). According to the 2017 National Population Projections of the United States Census Bureau, the year 2030 marks a demographic milestone by which one in five citizens will be older than 65 years (12, 13). Previous research has shown that certain physical and mental comorbidities particularly prevalent in the elderly could increase the risk of complications following total knee arthroplasty (TKA) and THA (3, 14).

Total joint replacement usually improves health-related quality of life; however, when the improvement is insignificant, the role of comorbidities is often emphasized (15, 16). The prevalence of comorbidities increases with age. It is estimated that between 60% and 88% of people aged 65 and older have at least one comorbidity (17), suggesting that a significant proportion of arthroplasty patients have comorbidities (18). In a large US study using administrative data, 83.7% of patients who underwent TKA or THA had at least one comorbidity (19), a rate higher than in the general population. In 2012, only 49.8% of adults in the US suffered from at least one comorbidity (20). Therefore, the comorbidities of patients undergoing joint replacement should receive more attention.

In some studies (21–27), higher comorbidity rate was associated with poorer joint replacement postoperative outcomes; in some, it had a small overall effect (28), and in some, it had no effect (29–33). Additionally, changes in postoperative comorbidities occurring in most patients over time are expected to have a dynamic impact on THA postoperative outcomes. Therefore, we aimed to find through THA postoperative follow-up whether preexisting and changes in comorbidities were associated with decreased postoperative physical function and poor pain outcomes.

## Materials and Methods

### Study Population

We selected the target population from our hospital according to the following selection criteria. The inclusion criteria were as follows: (1) consecutive patients who had to undergo primary unilateral THA between 2017 and 2019 and were willing to cooperate in completing outpatient follow-up after the THA procedure, and (2) the first language was Chinese to better understand the questionnaire. All enrolled patients provided informed consent. All surgical procedures were performed by the chief physician at our hospital.

Exclusion criteria: (1) patients with coagulation disorders or lower extremity venous thrombosis; (2) patients with local foci of infection or other diseases affecting postoperative hip function assessment; (3) patients with psychiatric disorders; (4) patients with bilateral THA.

All patients underwent a posterolateral approach with DePuy or Stryker implants (cementless or cemented). Cemented implants were used when intraoperative cortical thinness was determined, and the stability of the cementless prosthesis was considered insufficient. Otherwise, all patients received the same treatment regimen that included intraoperative antibiotic prophylaxis, prevention of thromboembolism complications, abduction pads, and postoperative full weight bearing. Perioperative multimodal pain management comprised of preoperative preemptive analgesia, intraoperative local infiltration anesthesia, and postoperative opioid-sparing analgesia. Patients could stand as of the first day after surgery, as the general circumstances permitted. A physical therapist guided their resumption of walking while teaching them how to avoid positions that could contribute to dislocation.

### Predictors and Their Definitions

The enrolled patients were divided into five age groups: ≤40, 40–50, 50–60, 60–70, and ≥70 years. The 36-item Short Form health survey (SF-36) body pain (BP) and physical function (PF) scales and Western Ontario and McMaster Universities Osteoarthritis Index (WOMAC; BP and PF) assessed the primary outcome before surgery and 3, 6, 12, and 24 months after surgery.

Comorbidity information was collected through patient self-reported history. The medical records were collected with the patients consent, and any relevant information was retrieved to guarantee the self-reported psychiatric history and diagnostic status accuracy; changes in comorbidities were collected at different follow-up time points. The SF-36 and WOMAC scales were completed at each follow-up visit. The preoperative diagnosis, preoperative comorbidities, waiting time before surgery, complications, 30-day readmission rate, and other factors were retrospectively studied by reviewing the hospital records.

Comorbidities were evaluated using the Charlson comorbidity index (CCI), which was originally used to predict mortality. However, CCI is commonly used to evaluate comorbidity in orthopedic patients because of its good prognostic value in terms of revision surgery, mortality, and various medical complications (9, 34–36). The diagnoses of all 19 comorbidities recorded were confirmed by the International Classification of Diseases, 10th revision (ICD-10) (37).

The patients were divided into four groups based on their comorbidity burden scores: CCI-0, no comorbid conditions; CCI-1 and CCI-2, comorbid conditions equal to a score of 1 and 2, respectively; CCI ≥ 3, comorbid conditions equal to a score of 3 or higher (38).

### Outcomes of Interest

The WOMAC is a widely used self-administered pain and functional ability scale for patients (39). It assesses pain, physical function, and stiffness, and asks patients about pain or difficulty in doing various daily activities, rated on a five-point scale from “none” to “extreme.” Scores for each subscale and total scores ranged between 0 and 100, with a higher score indicating worse pain, function, and stiffness. WOMAC BP and PF scores were collected and used to evaluate the THA postoperative pain and function (16).

The SF-36 is a generic health status measure, scored between 0 and100, with 100 being the best score. The SF-36 is composed of eight subscales: BP, PF, role physical (RP), role emotional (RE), social functioning (SF), mental health (MH), vitality (VT), and general health (GH). The postoperative SF-36 BP and PF scores were collected. SF-36 and WOMAC were shown to be effective and reliable (40).

The surgical complications were defined as dislocation, periprosthetic fracture, or surgical site infection (SSI) requiring surgical revision. Medical complications included those that were not life threatening, such as deep venous thrombosis, and those that were life threatening, such as myocardial infection, acute mesenteric ischemia, stroke, pulmonary embolism (PE), or any other ailment requiring a stay in the intensive care unit (41).

### Statistical Analyses

We examined SF-36 PF, SF-36 BP, WOMAC PF and WOMAC pain subscale scores as continuous outcome variables. Model diagnostics including Q-Q plots for residuals and Q-Q plots for random effects were tested. Based on the inherent skewness evident on these plots, we used gamma distribution for response with log link for these continuous variables. We used random intercept gamma generalized linear mixed model with log link to examine the association of increasing comorbidity score (Charlson and three indices from our novel comorbidity measure) with worsening QOL, as measured by the WOMAC and SF-36 PF and pain subscale scores, in the subsequent intervals (see above), that controlled for repeated observations. These reported effects were adjusted for age, baseline respective QOL score and the length of time from index THA. We present beta coefficients (ß) and *p*-values for these associations. The composition of preoperative diagnosis in different CCI groups was analyzed by chi-square test, and the influence of different preoperative diagnosis on postoperative function in different CCI groups was analyzed by one-way variance analysis.

## Results

### Clinical Characteristics of the Study Population

We recruited 468 consecutive patients who underwent unilateral THA at our department between 2017 and 2019. All patients were followed up for 24 months, except two who died after one year. Information on the five age groups is shown in Table 1. Preoperative CCI was positively associated with age, while postoperative CCI in the 60–70 and ≥70 years age groups increased with time over the two-year follow-up. Specific information on the preoperative CCI grouping is shown in Table 2. All patients completed the SF-36 (BP, PF) and WOMAC (BP, PF) surveys during all follow-up visits. The composition of preoperative diagnosis in different CCI groups (Supplementary Table S1). The composition of ONFH, Primary osteoarthritis, Fracture of femoral neck, Legg-Calve-Perthes disease and Ankylosing spondylitis in preoperative diagnosis had statistically significant differences in different subgroups. The effect of different preoperative diagnosis in different CCI groups on postoperative function (Supplementary Tables S2–S5). Supplementary Table S2 shows that different pre-op diagnosis could effect the functions of Group(pre-op CCI = 0) especially in the measurement scale of SF-36-BP. Supplementary Table S3 shows that the effect of pre-op diagnosis on the functions of Group(pre-op CCI = 1) was statistically significant different in the all four measurement scales. The statistically significant differences of the effect of pre-op diagnosis on the functions of Group(pre-op CCI = 2) were mainly reflected in measurement scale of womac-BP and SF-36-PF(showed in Supplementary Table S4). While in the Group((pre-op CCI ≥ 3), the statistically significant differences was showed in the measurement scale of SF-36-BP and SF-36-PF(Supplementary Table S5).

**Table 1:** Changes of preoperative CCI in different age groups.

	Age <40	40 ≤ age < 50	50 ≤ age < 60	60 ≤ age < 70	Age ≥70
*n*	44	81	101	127	115
gender(F/M)	24/20	50/31	55/46	71/56	63/52
BMI	22.62 ± 3.29	23.31 ± 2.94	23.62 ± 2.83	23.50 ± 3.26	23.22 ± 2.91
CCI					
Preop	0.29 ± 0.469	0.49 ± 0.543	1.26 ± 0.997	2.16 ± 0.791	3.85 ± 1.22
Post-op-3M	0.29 ± 0.469	0.49 ± 0.543	1.26 ± 0.997	2.24 ± 0.253	4.16 ± 1.27
Post-op-6M	0.29 ± 0.469	0.49 ± 0.543	1.26 ± 0.997	2.46 ± 0.853	4.65 ± 1.33
Post-op-1Y	0.29 ± 0.469	0.49 ± 0.543	1.26 ± 0.997	2.71 ± 1.02	5.19 ± 1.30
Post-op-2Y	0.29 ± 0.469	0.49 ± 0.543	1.28 ± 1.021	2.76 ± 1.058	5.42 ± 1.515

**Table 2:** Changes of WOMAC and SF-36 in different CCI groups.

	Preop CCI = 0	Preop CCI = 1	Preop CCI = 2	Preop CCI ≥ 3	*p*
*n*	101	113	105	149	
Age	45.28 ± 7.91	55.16 ± 7.98	62.76 ± 5.44	70.1 ± 7.72	<0.05
BMI	23.44 ± 3.16	23.10 ± 2.71	23.83 ± 3.18	23.27 ± 3.04	>0.05
womac-BP					
Preop	13.18 ± 1.937	19.00 ± 2.441	24.28 ± 3.164	32.71 ± 4.618	<0.05
Post-op-3M	5.08 ± 0.862	9.33 ± 2.159	16.01 ± 3.260	25.27 ± 5.171	<0.05
Post-op-6M	4.07 ± 0.750	6.31 ± 1.775	11.66 ± 3.815	21.22 ± 5.42	<0.05
Post-op-1Y	3.25 ± 0.907	4.78 ± 1.63	9.77 ± 3.08	20.40 ± 5.2	<0.05
Post-op-2Y	2.79 ± 0.88	4.65 ± 1.70	10.02 ± 3.29	19.27 ± 5.60	<0.05
womac-PF					
Preop	42.43 ± 2.101	46.44 ± 1.49	50.09 ± 2.932	66.32 ± 9.376	<0.05
Post-op-3M	24.61 ± 2.246	30.13 ± 2.979	37.20 ± 3.212	57.07 ± 9.973	<0.05
Post-op-6M	22.15 ± 2.064	26.62 ± 2.236	33.26 ± 4.121	51.60 ± 10.299	<0.05
Post-op-1Y	20.13 ± 2.506	24.99 ± 2.796	31.41 ± 3.919	51.29 ± 10.15	<0.05
Post-op-2Y	20.66 ± 2.03	23.12 ± 2.89	32.94 ± 3.64	51.23 ± 10.21	<0.05
SF-36-BP					
Preop	50.74 ± 4.00	43.31 ± 6.45	38.86 ± 5.16	31.57 ± 7.88	<0.05
Post-op-3M	73.03 ± 4.42	73.26 ± 9.26	55.12 ± 7.01	48.29 ± 9.23	<0.05
Post-op-6M	74.46 ± 3.52	72.62 ± 4.77	69.61 ± 5.12	62.73 ± 7.86	1 vs 2: *p* = 0.058; others *p* < 0.05
Post-op-1Y	72.66 ± 2.71	70.49 ± 4.65	68.22 ± 4.52	61.95 ± 9.27	<0.05
Post-op-2Y	71.95 ± 9.12	70.60 ± 4.83	68.57 ± 6.18	59.76 ± 9.13	1 vs 2: *p* = 0.261; 2 vs 3: *p* = 0.051: others *p* < 0.05
SF-36-PF					
Preop	51.93 ± 3.70	42.35 ± 5.78	34.39 ± 4.14	25.25 ± 5.69	<0.05
Post-op-3M	79.66 ± 4.01	66.57 ± 11.3	45.69 ± 8.13	32.42 ± 8.85	<0.05
Post-op-6M	80.57 ± 3.68	68.54 ± 9.62	53.32 ± 8.03	39.6 ± 8.68	<0.05
Post-op-1Y	81.2 ± 3.59	68.39 ± 10.02	52.08 ± 8.28	37.21 ± 8.96	<0.05
Post-op-2Y	80.79 ± 3.39	68.47 ± 9.56	53.23 ± 7.96	38.60 ± 9.60	<0.05
Days of preop	2.41 ± 0.739	2.97 ± 0.65	3.8 ± 0.526	5.01 ± 0.707	<0.05
Days of post-op	4.13 ± 0.67	4.89 ± 0.800	5.12 ± 0.73	5.97 ± 0.791	<0.05
Types of prosthesis					
cemented / cementless	101/0	113/0	103/2	145/4	
Complications					
Dislocation	0	0	1	3	
infection	0	0	0	2	
Periprosthetic fracture	0	0	1	1	
Venous thrombosis	0	0	0	1	
30 days readmission	0	0	1	3	
Number of deaths in 1 year	0	0	0	2	

### Changes in Comorbidities During Follow-Up

The preoperative CCI in the five age groups (<40, 40–50, 50–60, 60–70, and ≥70 years) was found to increase with age (0.29 ± 0.47, 0.49 ± 0.54, 1.26 ± 1.00, 2.16 ± 0.79, and 3.85 ± 1.22, respectively). Postoperative CCI increased in two age groups (60–70 and ≥70 years). In the 60–70 years group: 2.16 ± 0.79, 2.24 ± 0.25, 2.46 ± 0.85, 2.71 ± 1.02, and 2.76 ± 1.06, and the ≥70 age group: 3.85 ± 1.22, 4.16 ± 1.27, 4.65 ± 1.33, 5.19 ± 1.30, and 5.42 ± 1.52. There were no further CCI changes in the other three age groups during the two years of postoperative follow-up. Among them, the older the patients, the greater the postoperative CCI increase and the faster the physical state deteriorated (Table 1). And preoperative diagnosis, preoperative comorbidities was showed in Table 2.

### Changes in Pain and Functional Scores During Follow-Up

**Figure 1:**
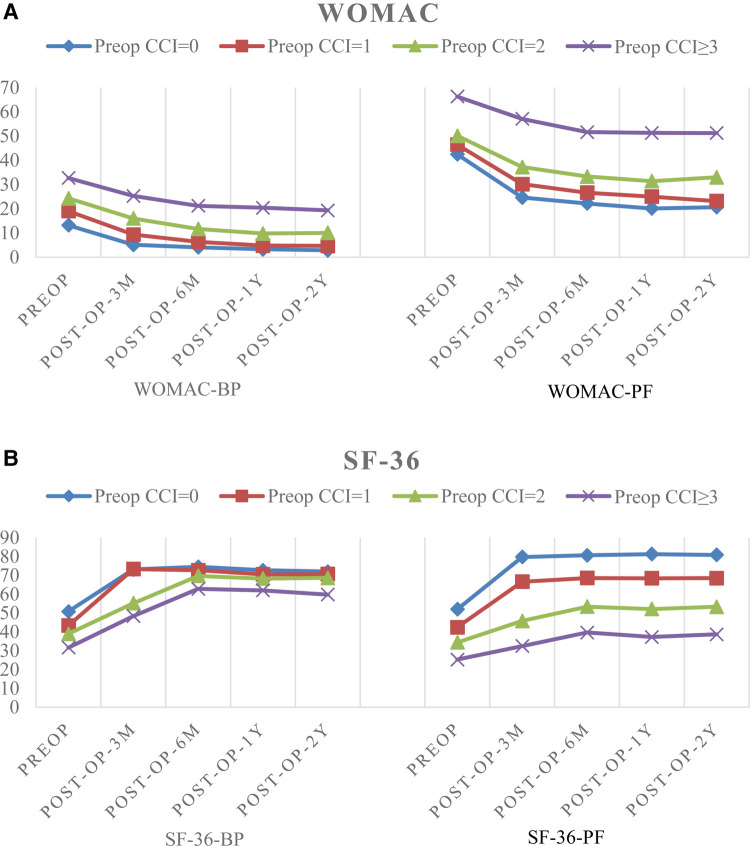
(**A**) The trend of changes in pain and functional scores based on WOMAC. (**B**) The trend of changes in pain and functional scores based on SF-36.

The baseline average WOMAC levels before surgery in the four CCI groups (CCI = 0, 1, 2, and CCI ≥ 3) were 13.18 ± 1.94, 19.00 ± 2.44, 24.28 ± 3.16, and 32.71 ± 4.62, respectively, for BP, and 42.43 ± 2.10, 46.44 ± 1.49, 50.09 ± 2.93, and 66.32 ± 9.38, respectively, for PF. The respective average baseline SF-36 levels of PF and BP were 51.93 ± 3.70, 42.35 ± 5.78, 34.39 ± 4.14, and 25.25 ± 5.69, and 50.74 ± 4.00, 43.31 ± 6.45, 38.86 ± 5.16, and 31.57 ± 7.88, respectively. The changes in values of CCI, WOMAC and SF-36 scores at the 3, 6, 12, and 24-month follow-up evaluations are shown in Tables 3 and 4, and the trend of the changes is shown in Figure 1. The WOMAC and SF-36 scores reached their maximum improvement three months after the operation. Subsequently, the improvement in the WOMAC score gradually decreased. The SF-36 score reached its maximum in CCI = 0 and CCI = 1 three months after surgery. The maximum was reached six months after the surgery in older patients (CCI = 2; CCI ≥ 3). We found no difference in the SF-36 scores between the 1- and 2-year follow-up assessments (*p* > 0.01). The WOMAC score in the CCI = 2 group had even rebounded during the 2-year follow-up.

**Table 3:** Clinical characteristics of different groups of preoperative CCI.

	Pre-op CCI = 0	Pre-op CCI = 1	Pre-op CCI = 2	Pre-op CCI ≥ 3	*p*
*n*	101	113	105	149	
Age	45.28 ± 7.91	55.16 ± 7.98	62.76 ± 5.44	70.1 ± 7.72	<0.001
BMI	23.44 ± 3.16	23.10 ± 2.71	23.83 ± 3.18	23.27 ± 3.04	>0.05
**Pre-operative diagnosis**					
ONFH	39	48	32	12	
DDH	23	19	15	18	
Primary osteoarthritis	8	17	38	69	
Rheumatoid arthritis	13	14	9	26	
Fracture of femoral neck	2	5	7	24	
Legg-Calve-Perthes disease	8	6	2	0	
Ankylosing spondylitis	8	4	2	0	
**Pre-operative comorbidities**					
Myocardial infarction	0	5	9	19	
Congestive Heart Failure	0	8	16	23	
Peripheral vascular disease	0	16	13	31	
Cerebrovascular disease	0	7	15	29	
Chronic Lung Disease	0	25	32	79	
Peptic ulcer	0	13	27	39	
Mild liver disease	0	21	11	49	
Diabetes mellitus (without comorbidities)	0	18	17	36	
paralysis	0	0	5	17	
Moderate to severe kidney disease	0	0	14	27	
Diabetes mellitus (with comorbidities)	0	0	16	47	
Moderate to severe liver disease	0	0	0	26	
HIV	0	0	0	2	

**Table 4:** Changes of WOMAC and SF-36 in different CCI groups during follow-up.

	Preop(Group 1)	Post-op-3M (Group 2)	Post-op-6M (Group 3)	Post-op-1Y (Group 4)	Post-op-2Y (Group 5)	*p*
**CCI**						
Preop CCI = 0	0 ± 0.00	0 ± 0.00	0 ± 0.00	0 ± 0.00	0 ± 0.00	
Preop CCI = 1	1 ± 0.00	1 ± 0.00	1.08 ± 0.27	1.12 ± 0.36	1.18 ± 0.49	1 vs 4: *p* = 0.007; 1 vs 5: *p* < 0.001; 2 vs 4: *p* = 0.007; 2 vs 5: *p* < 0.001; 3 vs 5: *p* = 0.013
Preop CCI = 2	2 ± 0.00	2 ± 0.00	2.1 ± 0.31	2.3 ± 0.54	2.55 ± 0.73	1 vs 4: <0.001; 1 vs 5: *p* < 0.001; 2 vs 4: *p* < 0.001; 2 vs 5: *p* < 0.001; 3 vs 4: *p* = 0.001; 3 vs 5: *p* < 0.001; 4 vs 5: *p* < 0.001
Preop CCI = 3	3.82 ± 0.91	3.82 ± 0.91	3.98 ± 1.07	4.35 ± 1.19	4.97 ± 1.50	1 vs 4: *p* < 0.001; 1 vs 5: *p* < 0.001; 2 vs 4: *p* < 0.001; 2 vs 5: *p* < 0.001; 3 vs 4: *p* = 0.012; 3 vs 5: *p* < 0.001; 4 vs 5: *p* < 0.001
**WOMAC-pain**						
Preop CCI = 0	13.18 ± 1.937	5.08 ± 0.862	4.07 ± 0.750	3.25 ± 0.907	2.787 ± 0.8778	<0.05
Preop CCI = 1	19.00 ± 2.441	9.33 ± 2.159	6.31 ± 1.775	4.79 ± 1.63	4.65 ± 1.70	4 vs 5: *p* = 0.628
Preop CCI = 2	24.28 ± 3.164	16.01 ± 3.260	11.66 ± 3.815	9.77 ± 3.08	10.02 ± 3.29	4 vs 5: *p* = 0.059;
Preop CCI ≥ 3	32.71 ± 4.618	25.27 ± 5.171	21.22 ± 5.42	20.40 ± 5.20	19.27 ± 5.60	3 vs 4: *p* = 0.223; 4 vs 5: *p* = 0.96
**WOMAC-PF**						
Preop CCI = 0	42.43 ± 2.101	24.61 ± 2.246	22.15 ± 2.064	20.13 ± 2.506	20.656 ± 2.0321	4 vs 5: *p* = 0.188
Preop CCI = 1	46.44 ± 1.49	30.13 ± 2.979	26.62 ± 2.236	24.99 ± 2.796	23.12 ± 2.89	3 vs 5: *p* = 0.176;
Preop CCI = 2	50.09 ± 2.932	37.20 ± 3.212	33.26 ± 4.121	31.41 ± 3.919	32.94 ± 3.64	3 vs 5: *p* = 0.526;
Preop CCI ≥ 3	66.32 ± 9.376	57.07 ± 9.973	51.60 ± 10.299	51.29 ± 10.15	51.23 ± 10.21	3 vs 4: *p* = 0.816;3 vs 5: *p* = 0.776;4 vs 5: *p* = 0.959
**SF-36-PF**						
Preop CCI = 0	51.93 ± 3.70	79.66 ± 4.06	80.57 ± 3.68	81.197 ± 3.59	80.787 ± 3.39	2 vs 3: *p* = 0.171; 2 vs 5: *p* = 0.091;3 vs 4: *p* = 0.352; 3 vs 5: *p* = 0.75; 3 vs 5 = 0.54
Preop CCI = 1	42.35 ± 5.78	66.57 ± 11.3	68.54 ± 9.62	68.39 ± 10.02	68.473 ± 9.556	3 vs 4: *p* = 0.913;4 vs 5: *p* = 0.95
Preop CCI = 2	34.39 ± 4.14	45.69 ± 8.13	53.32 ± 8.03	52.08 ± 8.28	53.23 ± 7.96	3 vs 4: *p* = 0.227;3 vs 5: *p* = 0.927;4 vs 5: *p*= 0.265
Preop CCI ≥ 3	25.25 ± 5.69	32.42 ± 8.85	39.6 ± 8.68	37.21 ± 8.96	38.60 ± 9.60	3 vs 5:0.363;4 vs 5: *p* = 0.207
**SF-36-BP**						
Preop CCI = 0	50.74 ± 4.00	73.03 ± 4.42	74.46 ± 3.52	72.66 ± 2.71	71.95 ± 9.12	2 vs 3: *p* = 0.135;2 vs 4: *p* = 0.692;2 vs 5: *p* = 0.257; 3 vs 4: *p* = 0.059; 3 vs 5: *p* = 0.009; 4 vs 5 = 0.46
Preop CCI = 1	43.31 ± 6.45	73.26 ± 9.26	72.62 ± 4.77	70.50 ± 4.65	70.60 ± 4.3	4 vs 5: *p* = 0.907
Preop CCI = 2	38.86 ± 5.16	55.12 ± 7.01	69.61 ± 5.12	68.22 ± 4.52	68.57 ± 5.18	3 vs 4: *p* = 0.066;3 vs 5: *p* = 0.169;4 vs 5: *p* = 0.64
Preop CCI ≥ 3	31.57 ± 7.88	48.29 ± 9.23	62.73 ± 7.86	61.95 ± 9.27	59.76 ± 9.13	3 vs 4: *p* = 0.489;4 vs 5 = 0.052

### Increased Comorbidities Were Associated with Subsequent Pain and Functional Changes

As shown in Table 5, the postoperative deterioration in the CCI score was correlated with WOMAC scores (PF, ß = 0.164; *p* < 0.01; BP, β = 0.277; *p* < 0.001; the lower the score, the better), and SF-36 (PF, ß = −0.091; *p* < 0.002; BP, β = −1.186; *p* = 0.323; the higher the score, the better).

**Table 5:** Relationship between postoperative CCI and postoperative pain and functional measurements after THA.

	β-coefficient (95% CI);	*p*-value
	CCI	
WOMAC-BP	0.277 (0.257, 0.297)	*p* < 0.001
WOMAC-PF	0.164 (0.156, 0.172)	*p* < 0.001
SF-36-PF	−0.091 (−0.099, −0.084)	*p* < 0.001
SF-36-BP	−1.186 (−0.013, 0.004)	*p* = 0.323

### Effects of CCI on Preoperative Waiting Time, and Rates of Complications, 30-Day Readmission, and Mortality

We found that the preoperative waiting time in the four CCI groups (CCI = 0, 1, 2, and CCI  ≥  3) were 2.41 ± 0.74, 2.97 ± 0.65, 3.80 ± 0.53, and 5.01 ± 0.71 days, respectively. We found no complications during follow-up in the CCI = 0 and 1 groups, 1 joint dislocation and 1 periprosthetic fracture in the CCI = 2 group, respectively, and 3 joint dislocations, 2 postoperative infections, 1 periprosthetic fracture, and 1 venous thrombosis in the CCI ≥ 3 group. The total complications rate during the follow-up period was 1.92%, and it was 4.69% in the CCI ≥ 3 group. The overall 30-day readmission rate was 0.85%, and it was 2.01% in the CCI ≥ 3 group. The overall mortality rate within one year was 0.43%. It was 1.34% in the CCI ≥ 3 group, the only group in which mortality was recorded.

## Discussion

Joint replacement was shown to reduce pain, enhance function, and improve the quality of life (16, 42). In the US, approximately 300,000 THA procedures are performed every year, and the demand for these surgeries is expected to grow (43). Therefore, it is particularly important to understand the factors that affect THA surgery outcomes. Surgical and prosthetic techniques are perfected continually, making us pay attention to the comorbidities preoperatively. The CCI can help assess the patients burden of comorbidities preoperatively. These comorbidities inevitably affect the postoperative recovery. Studying their impact on the patients level of pain and function can better guide their reasonable expectations and exercise routine. We dynamically followed the CCI trends of patients at different ages and the changes in function and pain during the follow-up period in patients with different CCI levels.

Advanced age is associated with more comorbidities and disabilities before surgery. Previous studies have found that age is an important predictor of moderate-to-severe activity limitation after TKA (44). The prevalence of comorbidities increases with age. It is estimated that 60%–88% of people aged 65 years and over have at least one comorbidity (17). It is also estimated that a large proportion of patients with joint replacements have comorbidities (18). We found a positive association between the THA patients age and preoperative baseline CCI. This might be a concern since age could be used as a proxy for a higher comorbidity load. However, because neither the distribution of comorbidity groups nor the distribution of age groups changed over time, we interpret the findings as a clear link between comorbidity load and function scores, rather than a link between age and function scores. Age alone has no bearing on the result of joint arthroplasty and should not be used as a criterion for deciding who should have the procedure (31). Through follow-up, we also found that the older the patient, the greater the increase in CCI following the surgery. We found statistically significant differences in functional scores at different follow-up time points for different preoperative diagnoses in different subgroups, which could be attributed to the different baseline functions of patients with different diagnoses. Also patients with different preoperative diagnoses had different perceptions of their physical condition, which led to different expectations and motivation to participate in rehabilitation, thus influencing the functional scores at different follow-up points.

Several studies have shown that pain and satisfaction after THA are affected by preoperative comorbidities (45–47). However, using three comorbidity indexes, including CCI, Greene et al. (48) found only a marginal association between preoperative comorbidity burden and patient-reported health-related quality of life (HRQoL). All patients, regardless of the comorbidity burden, showed a significant improvement in HRQoL after the surgery. During that study 3-month follow-up, a positive correlation was found between the comorbidity burden in THA patients and their HRQoL gain. We found that the improvement in the patients SF-36 and WOMAC scores by three months after the surgery was affected by their preoperative CCI level. The higher the preoperative CCI, the lower and slower the improvement. We noticed that research on the effects of comorbidities on THA postoperative function and satisfaction is inconsistent. This inconsistency also shows that CCI influence on THA postoperative function varies with time, and its influence level differs between time periods.

We also found that WOMAC and SF-36 pain and function scores improved significantly from the preoperative to the postoperative assessment. Most of the patients showed the most significant improvement at 3, or 6 months after surgery, probably because the patients overcame the psychological impact of the surgery and were able to be more active in their own rehabilitation and participation in life work as the pain level decreased or disappeared. However, this improvement was not sustained during the 2-year follow-up after surgery. Patients with different preoperative CCI scores had different degrees and rates of functions and pain recovery after surgery. Comorbidities were related to a gradual deterioration of function and pain outcomes after THA.

In a study in Australia, the baseline comorbidities of patients undergoing THA or TKA predicted the change in the SF-36 score 12 months after the surgery (49). A study of 551 TKA patients showed that the SF-36 and WOMAC scores gradually decreased over the years after the initial postoperative improvement (27). This decline was related to the preoperative comorbidities baseline (50). We obtained similar results. This could be explained by the increase in CCI with age, which affected the SF-36 and WOMAC scores.

Our study found that the patients postoperative complications change over time, and the older the patient, the greater the CCI increase. This explains why older patients after THA do not continue to improve. This knowledge could also guide clinicians monitoring and early interventions aimed to improve the THA patients long-term quality of life by reducing the postoperative impact of comorbidities on pain and functional results.

Comorbidities in cancer patients are associated with mortality, length of hospital stay, postoperative complications, progression-free survival rate, and disability (51, 52). This association has been verified in various situations, including pneumonia, heart disease, spinal surgery, and amputation (52). Previous studies have shown that delayed surgery increases postoperative complications, mortality, and costs of other orthopedic surgeries (53–58). Surgical delay after admission for elective THA affects the related total hospitalization costs. The delayed surgery rate in elective primary THA after admission was 2.31%, with a median operative delay of two days (range, 1–26 days) (59). We found that an increase in CCI was associated with a longer waiting time before surgery and a higher incidence of complications.

Researchers found that for every one-point increase in CCI, the risk of delayed THA surgery increased by 52% (59). The surgical complication rate was higher in the delayed operation group, and included superficial surgical site infection, inter-organ infection, wound dehiscence, and reoperation. Medical complications in the delayed surgery group were also higher and included pneumonia, unplanned intubation, renal insufficiency, urinary tract infection, blood transfusion, and sepsis. We also found that the preoperative waiting time was significantly longer in patients with CCI ≥ 3, and their postoperative complication rate was higher than the other groups (59). The main reason for the long preoperative waiting time was the need to optimize the patient’s biologic status before surgery and to manage comorbidities in order to better cope with the surgery and postoperative recovery.

The exponential increase in surgeries has increased the probability of postoperative complications for overall patients, including surgical site infection, sepsis, joint dislocation, and revision arthroplasty. These complications increased the length of hospital stay and readmission rate (60). Previous studies have found that CCI-2 could be used to predict surgical site infection after joint replacement (61).

Reducing the incidence of THA delays without reducing the quality of treatment might be an important strategy for optimizing surgical efficiency and reducing costs. Our study identified the risk factors that affect delayed THA surgery, striving to better understand and manage these risk factors in THA patients.

Hospital readmission is used as an indicator of the quality of care (62). The waste and cost of medical resources for readmission is high (18), and CCI can be used to assess this risk after arthroplasty, hand and upper limb surgery, spinal surgery, and trauma surgery (9). We found that the 30-day readmission rate was significantly higher in patients with CCI ≥ 3.

The risk of all-cause mortality after THA and TKA decreased from 1% in 1997 to 0.6% in 2011 (36). This could be due to the introduction of enhanced rehabilitation programs and minimally-invasive surgery, multimodal postoperative pain management, and early return to activity, all of which were reported to affect mortality after THA and TKA (36, 63, 64). When the comorbidity burden during THA and TKA surgeries was moderate or high, the mortality risk did not decrease (38).

Although the mortality rate after joint replacement is very low, studies have found that it increases by comorbidities (65, 66). Kreder et al. (67) found that the mortality rate of patients with comorbidities was 24 times higher than in patients without comorbidities. Other authors have found that CCI > 1 was associated with higher mortality risk within two years after sustaining proximal femoral fracture (68). Our study found that the overall 2-year mortality rate after THA was not high, but it was significantly higher in patients with CCI ≥ 3.

### Limitations

We acknowledge that our study has several limitations: First, only patients with primary unilateral THA were included; patients with revision and bilateral THA were excluded. Second, the number of patients was small. Third, the duration of follow-up was short. We will continue to follow these patients to obtain long-term results and further evaluate the impact of complications. Fourth, the use of CCI as a measure of comorbidity might have limitations. It was developed to quantify the impact of comorbidities on mortality, and it was validated in breast cancer patients but not in THA patients. Although this indicator has been widely used in orthopedic research, its suitability might still affect its effectiveness. Fifth, another limitation of using CCI is that the only mental illness it considers is dementia (69). Sixth, cementless and cemented implants were used in the patient cohort, although there are fewer cemented prostheses, different fixation methods can have an impact on postoperative outcomes. Seventh, there were 30-day readmissions only in the CCI = 2, CCI ≥ 3 groups during follow-up, as well as deaths only in the CCI ≥ 3 group and in small numbers, with some shortcomings in the guidance. Finally, the preoperative diagnoses of the included patients were not consistent, and different preoperative diagnoses would effect preoperative CCI and postoperative outcomes to some extent.

## Conclusions

We found that comorbidities were associated with worse physical function and pain outcomes after THA. A higher CCI score in THA patients was associated with longer preoperative waiting time and postoperative function and pain recovery time, a lower degree of recovery, and higher rates of complications, 30-day readmission, and mortality. These impacts were greater in the older age group. Understanding this information allows us to place greater emphasis on the management of perioperative comorbidities, guide postoperative recovery, and provide more realistic expectations for our patients.

## Data Availability

The original contributions presented in the study are included in the article/Supplementary Materials, further inquiries can be directed to the corresponding author/s.
