# Supplementary material for: Effects of Comorbidities on Pain and Function After Total Hip Arthroplasty
Source: Front Surg. 2022 May 11;9:829303. doi: 10.3389/fsurg.2022.829303 (PMC9130629; doi:10.3389/fsurg.2022.829303)
Supplement: Supplementary file 1 [file Table_1.docx]

Supplementary Table 1. The effect of pre-op diagnosis on pre-op CCI.

|  |  | Pre-op CCI=0 | Pre-op CCI=1 | Pre-op CCI=2 | Pre-op CCI≥3 | P |
| --- | --- | --- | --- | --- | --- | --- |
| ONFH | YES | 39 | 48 | 32 | 12 | <0.001 |
|  | NO | 62 | 65 | 73 | 137 |  |
| DDH | YES | 23 | 19 | 15 | 18 | 0.143 |
|  | NO | 78 | 94 | 90 | 131 |  |
| Primary osteoarthritis | YES | 8 | 17 | 38 | 69 | <0.001 |
|  | NO | 93 | 96 | 67 | 80 |  |
| Rheumatoid arthritis | YES | 13 | 14 | 9 | 26 | 0.224 |
|  | NO | 88 | 99 | 96 | 123 |  |
| Fracture of femoral neck | YES | 2 | 5 | 7 | 24 | <0.001 |
|  | NO | 99 | 108 | 98 | 125 |  |
| Legg-Calve-Perthes disease | YES | 8 | 6 | 2 | 0 | 0.004 |
|  | NO | 93 | 107 | 103 | 149 |  |
| Ankylosing spondylitis | YES | 8 | 4 | 2 | 0 | 0.004 |
|  | NO | 93 | 109 | 103 | 149 |  |

Supplementary Table 2. The effect of pre-op diagnosis on the function of Group(pre-op CCI=0).

|  | ONFH | DDH | Primary OA | RA | Fracture of femoral neck | Legg-Calve-Perthes disease | AS | P |
| --- | --- | --- | --- | --- | --- | --- | --- | --- |
| womac-BP-Preop | 14.08±1.81 | 12.46±1.63 | 12.83±2.71 | 12.20±1.48 | 15.00±0.00 | 11.75±1.71 | 13.67±1.63 | 0.037 |
| Post-op-3M | 5.00±0.95 | 5.09±0.70 | 4.83±0.75 | 5.20±0.92 | 5.00±0.00 | 4.75±0.96 | 5.67±0.82 | 0.661 |
| Post-op-6M | 3.96±0.82 | 4.09±0.70 | 4.17±0.75 | 4.20±0.79 | 5.00±0.00 | 4.00±0.82 | 4.00±0.63 | 0.875 |
| Post-op-1Y | 3.00±0.95 | 3.64±0.67 | 2.83±1.47 | 3.60±0.70 | 4.00±0.00 | 3.50±0.58 | 3.00±0.63 | 0.242 |
| Post-op-2Y | 2.52±0.67 | 3.09±0.83 | 2.67±1.37 | 3.00±0.94 | 4.00±0.00 | 3.00±0.82 | 2.67±1.03 | 0.392 |
| womac-PF-Preop | 43.61±2.21 | 41.18±1.40 | 42.17±2.64 | 42.40±1.35 | 45.00±0.00 | 42.25±2.63 | 44.00±2.28 | 0.167 |
| Post-op-3M | 24.96±2.42 | 23.55±2.34 | 25.84±2.48 | 34.20±2.04 | 24.00±0.00 | 24.25±0.96 | 25.00±2.00 | 0.497 |
| Post-op-6M | 22.22±2.41 | 21.36±1.80 | 23.17±2.79 | 21.80±1.55 | 23.00±0.00 | 22.25±1.50 | 22.67±1.51 | 0.706 |
| Post-op-1Y | 20.09±3.36 | 19.55±1.69 | 20.54±2.59 | 19.70±1.49 | 21.00±0.00 | 21.25±2.22 | 20.83±1.83 | 0.89 |
| Post-op-2Y | 20.22±2.49 | 19.36±0.92 | 20.83±1.47 | 20.90±1.10 | 23.00±0.00 | 22.25±1.50 | 22.67±1.51 | 0.011 |
| SF-36-BP-Preop | 53.09±4.01 | 51.00±1.73 | 45.67±1.50 | 49.20±4.10 | 48.00±0.00 | 47.75±3.20 | 51.33±2.50 | <0.001 |
| Post-op-3M | 74.30±3.96 | 73.45±3.61 | 68.33±4.26 | 71.20±2.47 | 65.00±0.00 | 72.75±6.70 | 76.17±4.17 | 0.01 |
| Post-op-6M | 74.48±3.89 | 73.55±1.81 | 71.50±2.74 | 73.50±2.59 | 74.00±0.00 | 77.50±2.38 | 78.67±3.01 | 0.004 |
| Post-op-1Y | 72.74±3.48 | 73.46±1.51 | 71.17±1.47 | 73.10±2.60 | 70.00±0.00 | 72.50±2.38 | 72.17±2.48 | 0.664 |
| Post-op-2Y | 73.04±3.96 | 67.91±2.62 | 72.17±2.56 | 73.40±2.88 | 70.00±0.00 | 72.50±2.38 | 72.50±2.43 | 0.845 |
| SF-36-PF-Preop | 51.30±3.83 | 53.36±3.30 | 54.17±1.47 | 48.80±4.94 | 50.00±0.00 | 52.75±1.26 | 54.50±2.26 | 0.013 |
| Post-op-3M | 79.65±2.85 | 81.36±2.20 | 82.00±2.44 | 76.40±7.43 | 79.00±0.00 | 80.75±2.22 | 79.00±2.83 | 0.083 |
| Post-op-6M | 80.39±2.48 | 81.73±2.10 | 83.00±1.41 | 78.00±7.15 | 79.00±0.00 | 81.50±1.73 | 80.67±2.42 | 0.163 |
| Post-op-1Y | 81.00±2.76 | 82.27±2.24 | 83.17±2.14 | 78.90±6.51 | 82.00±0.00 | 81.25±2.22 | 81.67±3.01 | 0.316 |
| Post-op-2Y | 81.57±2.11 | 81.18±2.56 | 82.17±1.83 | 77.90±6.23 | 78.00±0.00 | 80.75±1.50 | 81.00±2.68 | 0.104 |

Supplementary Table 3. The effect of pre-op diagnosis on the function of Group(pre-op CCI=1).

|  | ONFH | DDH | Primary OA | RA | Fracture of femoral neck | Legg-Calve-Perthes disease | AS | P |
| --- | --- | --- | --- | --- | --- | --- | --- | --- |
| womac-BP-Preop | 19.37±2.94 | 18.31±1.67 | 19.92±2.21 | 17.92±1.89 | 17.50±3.00 | 19.60±0.89 | 19.50±1.29 | 0.201 |
| Post-op-3M | 9.66±2.33 | 8.00±0.97 | 8.69±1.55 | 8.69±1.84 | 10.75±2.50 | 12.00±2.00 | 11.00±2.00 | 0.001 |
| Post-op-6M | 5.81±1.47 | 5.63±0.89 | 6.00±1.22 | 6.85±1.52 | 9.00±2.94 | 8.00±2.28 | 7.25±2.63 | <0.001 |
| Post-op-1Y | 4.53±1.39 | 4.06±0.93 | 4.07±1.19 | 5.00±1.78 | 7.00±2.45 | 6.80±1.30 | 7.00±1.15 | <0.001 |
| Post-op-2Y | 4.32±1.29 | 3.88±0.89 | 3.77±0.93 | 5.08±2.14 | 6.75±2.22 | 6.80±1.48 | 7.50±1.29 | <0.001 |
| womac-PF-Preop | 46.63±1.58 | 45.88±0.96 | 46.07±1.19 | 46.31±1.25 | 45.75±0.96 | 48.60±2.30 | 46.50±1.29 | 0.015 |
| Post-op-3M | 29.11±1.74 | 31.38±3.44 | 30.23±1.88 | 29.77±3.51 | 31.75±3.86 | 33.20±5.36 | 30.25±4.19 | 0.024 |
| Post-op-6M | 26.39±2.02 | 25.75±1.18 | 26.23±1.16 | 26.77±2.72 | 29.50±1.73 | 29.20±3.77 | 27.00±3.36 | 0.007 |
| Post-op-1Y | 25.55±1.97 | 23.94±2.35 | 23.23±3.75 | 24.69±2.46 | 25.75±2.5 | 26±4.47 | 28.5±2.89 | 0.009 |
| Post-op-2Y | 26.21±2.08 | 24.5±2.53 | 24.69±3.61 | 26.77±2.71 | 29.5±1.73 | 29.2±3.77 | 27±3.37 | 0.001 |
| SF-36-BP-Preop | 47.18±4.54 | 41.5±6.87 | 42.85±5.24 | 40.31±3.04 | 39.5±14.2 | 37.4±4.98 | 36.25±3.5 | <0.001 |
| Post-op-3M | 69.24±6.18 | 63.69±8.4 | 62.92±8.26 | 56.54±5.04 | 51.75±12.34 | 55.8±6.3 | 48.5±3.7 | <0.001 |
| Post-op-6M | 74.61±3.58 | 72.81±4.37 | 71.77±2.74 | 73.15±3.11 | 63.5±9.75 | 67.8±4.87 | 69.25±5.5 | <0.001 |
| Post-op-1Y | 72.16±3.37 | 72.75±3.02 | 69±2.16 | 70.54±3.73 | 60.75±10.37 | 67±2.92 | 64.5±4.04 | <0.001 |
| Post-op-2Y | 73.05±3.42 | 72.44±3.29 | 68.62±1.94 | 69.62±3.48 | 60.75±10.37 | 67±2.92 | 64±3.16 | <0.001 |
| SF-36-PF-Preop | 46.18±2.45 | 46.81±1.97 | 39.54±4.74 | 35.31±2.63 | 35.75±4.27 | 38.6±0.55 | 31.5±3.7 | <0.001 |
| Post-op-3M | 71.42±2.76 | 69.81±2.99 | 59.23±5.49 | 48.77±3.96 | 45.5±4.04 | 48.6±1.14 | 39.75±4.65 | <0.001 |
| Post-op-6M | 76.03±3.03 | 74.19±2.23 | 66.85±3.72 | 56.39±4.44 | 52.75±3.5 | 55.2±3.7 | 52.25±4.5 | <0.001 |
| Post-op-1Y | 76.08±3.11 | 75±2.1 | 67.23±4.11 | 56.23±3.81 | 52±4.16 | 54.8±3.77 | 49±5.89 | <0.001 |
| Post-op-2Y | 75.87±2.91 | 74.19±2.23 | 66.85±3.72 | 56.39±4.44 | 52.75±3.5 | 55±5.66 | 52.25±4.5 | <0.001 |

Supplementary Table 4. The effect of pre-op diagnosis on the function of Group(pre-op CCI=2).

|  | ONFH | DDH | Primary OA | RA | Fracture of femoral neck | Legg-Calve-Perthes disease | AS | P |
| --- | --- | --- | --- | --- | --- | --- | --- | --- |
| womac-BP-Preop | 23.61±3.28 | 23.4±3.09 | 24.13±2.93 | 26±2.18 | 27±4 | 25.67±2.89 | 24.5±2.12 | 0.082 |
| Post-op-3M | 16.45±2.96 | 14.87±2.53 | 14.5±2.69 | 17.78±2.64 | 19.43±4.04 | 20.67±3.06 | 19.5±0.71 | <0.001 |
| Post-op-6M | 11.77±3.06 | 9.53±2.77 | 10.05±3.05 | 14.44±4.33 | 17.43±2.57 | 17±1.73 | 15.5±0.71 | <0.001 |
| Post-op-1Y | 9.32±2.12 | 8.2±1.93 | 8.79±2.73 | 12.22±3.77 | 13.71±2.29 | 15±2 | 14.5±0.71 | <0.001 |
| Post-op-2Y | 10.03±2.58 | 8.27±2.74 | 9.21±3.43 | 12.33±3.81 | 13.86±2.12 | 12±1.73 | 11.5±0.71 | 0.001 |
| womac-PF-Preop | 49.81±2.83 | 49.8±3.08 | 50.18±3.27 | 50.56±1.81 | 50.43±2.3 | 50.67±3.06 | 50.5±6.36 | 0.989 |
| Post-op-3M | 36.81±2.99 | 36.73±3.06 | 37.53±3.57 | 37.11±2.26 | 36.29±2.81 | 39.33±3.21 | 41±5.66 | 0.445 |
| Post-op-6M | 32.45±3 | 32±4.99 | 33.32±3.39 | 36.67±5.7 | 34.43±5.06 | 34.67±8.08 | 32.5±3.54 | 0.132 |
| Post-op-1Y | 30.77±4.23 | 30.2±4.26 | 31.08±3.17 | 34±2.92 | 33.43±4.5 | 35±6.08 | 32.5±0.71 | 0.084 |
| Post-op-2Y | 32.45±3 | 32±4.99 | 33.32±3.39 | 34.22±3.67 | 33.57±4.43 | 33±5.2 | 32.5±3.54 | 0.778 |
| SF-36-BP-Preop | 40.61±5.34 | 38.13±6.14 | 38.71±4.95 | 37.56±1.33 | 35.43±5.71 | 40±4.36 | 36±2.83 | 0.213 |
| Post-op-3M | 57.32±7.93 | 55.6±9.19 | 54.68±5.69 | 53.33±0.71 | 51.14±7.03 | 54.67±8.74 | 48.5±0.71 | 0.245 |
| Post-op-6M | 71.71±4.53 | 69.93±5.96 | 69.5±4.26 | 68.78±2.39 | 64.43±6.9 | 67±8.66 | 62.5±2.12 | 0.006 |
| Post-op-1Y | 69.39±3.96 | 68.67±5.54 | 67.95±4.19 | 67.44±2.19 | 64.71±7.41 | 69.33±5.03 | 66±2.83 | 0.28 |
| Post-op-2Y | 70.13±4.95 | 69±6.5 | 69.05±4.49 | 65.22±1.64 | 64±6.19 | 69±5.2 | 62.5±2.12 | 0.015 |
| SF-36-PF-Preop | 38.1±4.03 | 32.93±2.52 | 34.08±1.95 | 33.89±2.37 | 29.43±1.81 | 25±1.73 | 27.5±0.71 | <0.001 |
| Post-op-3M | 55.68±6.48 | 44±3.27 | 42.82±1.92 | 40.44±4.1 | 37±2.38 | 31.33±2.52 | 33.5±0.71 | <0.001 |
| Post-op-6M | 63.55±6.29 | 51.07±3.47 | 49.61±2.27 | 48.78±4.15 | 46.71±2.69 | 40±1.73 | 46±0 | <0.001 |
| Post-op-1Y | 62.87±5.56 | 50.6±3.7 | 47.82±2.6 | 46.89±4.7 | 45±2.24 | 39.67±2.08 | 43.5±2.12 | <0.001 |
| Post-op-2Y | 63.32±6.27 | 51.2±3.55 | 49.47±2.23 | 48.78±4.15 | 46.71±2.69 | 40±1.73 | 46±0 | <0.001 |

Supplementary Table 5. The effect of pre-op diagnosis on the function of Group(pre-op CCI≥3).

|  | ONFH | DDH | Primary OA | RA | Fracture of femoral neck | Legg-Calve-Perthes disease | AS | P |
| --- | --- | --- | --- | --- | --- | --- | --- | --- |
| womac-BP-Preop | 33.61±2.64 | 30.9±2.81 | 32.28±5.71 | 34.79±3.19 | 32.8±5.32 |  |  | 0.088 |
| Post-op-3M | 25.83±3.82 | 22.45±3.97 | 24.81±6.22 | 28.26±3.6 | 26±4.16 |  |  | 0.009 |
| Post-op-6M | 20.56±3.75 | 18.5±4.45 | 21.21±6.64 | 23.74±3.71 | 22.47±4.41 |  |  | 0.034 |
| Post-op-1Y | 19.67±3.76 | 18.2±3.78 | 20.6±6.5 | 22.21±4.81 | 21.27±3.13 |  |  | 0.15 |
| Post-op-2Y | 19±4.79 | 17.65±3.77 | 18.85±7.06 | 21.21±4.47 | 20.6±4 |  |  | 0.278 |
| womac-PF-Preop | 69.72±12.5 | 65.8±9.31 | 64.4±8.89 | 69.16±6.98 | 65.33±8.49 |  |  | 0.177 |
| Post-op-3M | 60.28±13.66 | 56.9±10.81 | 54.7±9.19 | 60.16±6.91 | 56.93±8.32 |  |  | 0.174 |
| Post-op-6M | 54.56±13.63 | 50.9±12.61 | 49.49±9.16 | 54.26±6.46 | 52.2±9.28 |  |  | 0.303 |
| Post-op-1Y | 54.83±13.53 | 49.85±12.11 | 49.11±9.07 | 54.47±6.96 | 51.8±8.11 |  |  | 0.153 |
| Post-op-2Y | 53.83±12.94 | 50.6±12.86 | 49.17±9.1 | 54.26±6.46 | 51.53±9.48 |  |  | 0.306 |
| SF-36-BP-Preop | 32.22±11.37 | 32.65±7.41 | 32.28±7.22 | 32.11±5.47 | 26.47±7.11 |  |  | 0.121 |
| Post-op-3M | 50.39±14.34 | 50.15±8.09 | 48.83±8.34 | 48.37±6.49 | 41.47±5.99 |  |  | 0.036 |
| Post-op-6M | 62.28±10.13 | 65.65±8.52 | 63.28±6.97 | 63.47±6.44 | 56.73±5.68 |  |  | 0.015 |
| Post-op-1Y | 60.72±11.93 | 65.15±10.25 | 62.85±8.01 | 63.37±7.56 | 54.53±6.67 |  |  | 0.009 |
| Post-op-2Y | 60.72±11.21 | 65.25±10.19 | 58.53±7.77 | 60.74±7.12 | 53.87±7.64 |  |  | 0.004 |
| SF-36-PF-Preop | 28.72±9.13 | 27±5.14 | 26±4.39 | 21.95±2.7 | 20.6±1.8 |  |  | <0.001 |
| Post-op-3M | 39.78±15.01 | 34.9±7.34 | 33.04±6.04 | 26.84±3.11 | 25.4±2.56 |  |  | <0.001 |
| Post-op-6M | 46.89±14.89 | 40.5±7.47 | 39.98±6.26 | 35.95±3.5 | 33.07±3.47 |  |  | <0.001 |
| Post-op-1Y | 45.11±14.77 | 38.95±7.62 | 37.6±6.23 | 32.53±4.67 | 30.13±3.02 |  |  | <0.001 |
| Post-op-2Y | 44.28±17.57 | 38.7±9.67 | 39.6±6.61 | 35.32±4.03 | 32.67±3.72 |  |  | 0.004 |
